# Self-Net: Lifelong Learning via Continual Self-Modeling

**DOI:** 10.3389/frai.2020.00019

**Published:** 2020-04-09

**Authors:** Jaya Krishna Mandivarapu, Blake Camp, Rolando Estrada

**Affiliations:** Department of Computer Science, Georgia State University, Atlanta, GA, United States

**Keywords:** deep learning, continual learning, autoencoders, manifold learning, catastrophic forgetting

## Abstract

Learning a set of tasks over time, also known as continual learning (CL), is one of the most challenging problems in artificial intelligence. While recent approaches achieve some degree of CL in deep neural networks, they either (1) store a new network (or an equivalent number of parameters) for each new task, (2) store training data from previous tasks, or (3) restrict the network's ability to learn new tasks. To address these issues, we propose a novel framework, Self-Net, that uses an autoencoder to learn a set of low-dimensional representations of the weights learned for different tasks. We demonstrate that these low-dimensional vectors can then be used to generate high-fidelity recollections of the original weights. Self-Net can incorporate new tasks over time with little retraining, minimal loss in performance for older tasks, and without storing prior training data. We show that our technique achieves over 10X storage compression in a continual fashion, and that it outperforms state-of-the-art approaches on numerous datasets, including continual versions of MNIST, CIFAR10, CIFAR100, Atari, and task-incremental CORe50. To the best of our knowledge, we are the first to use autoencoders to sequentially encode sets of network weights to enable continual learning.

## 1. Introduction

Lifelong or continual learning (CL) is one of the most challenging problems in machine learning, and it remains a significant hurdle in the quest for artificial general intelligence (AGI) (Goodfellow et al., 2013; Kemker et al., 2018). In this paradigm, a single system must learn to solve new tasks without forgetting previously learned information. Different tasks might require different data (e.g., images vs. text) or they might process the same data in different ways (e.g., classifying an object in an image vs. segmenting it). Crucially, in CL there is no point at which a system stops learning; it must always be able to update its representation of its problem domain(s).

CL is particularly challenging for deep neural networks because they are trained end-to-end. In standard deep learning we tune all of the network's parameters based on training data, usually via backpropagation (Rumelhart et al., 1986). While this paradigm has proven highly successful for individual tasks, it is not suitable for continual learning because it overwrites existing weights (a phenomenon evocatively dubbed *catastrophic forgetting*; Robins, 1995). For example, if we first train a network on task A and then on task B, the latter training will modify the weights learned for A, thus likely reducing the network's performance on the first task.

**Figure 1:**
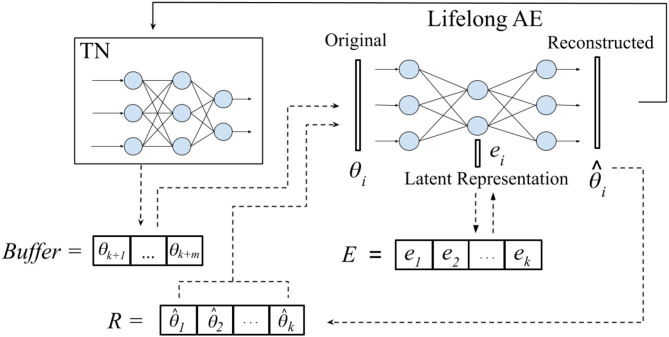
Framework overview. Our proposed system has a set of reusable *task-specific networks* (TN), a *Buffer* for storing the latest *m* tasks, and a lifelong, *auto-encoder* (AE) for long-term storage. Given new tasks {*t*_*k*+1_, …, *t*_*k*+*m*_}, where *k* is the number of tasks previously encountered, we first train *m* task-networks independently to learn {θ_*k*+1_, …, θ_*k*+*m*_} optimal parameters for these tasks. These networks are temporarily stored in the Buffer. When the Buffer fills up, we incorporate the new networks into our long-term representation by retraining the AE on both its approximations of previously learned networks and the new batch of networks. When an old network is needed (e.g., when a task is revisited), we reconstruct its weights and load them onto the corresponding TN (solid arrow). Even when the latent representation *e*_*i*_ is asymptotically smaller than θ_*i*_, the reconstructed network closely approximates the performance of the original.

In this paper, we propose a novel approach, Self-Net, that achieves continual learning by decoupling how it *learns* a new task from how it *stores* it. Specifically, we learn compressed representations of previously learned parameters in a continual fashion; these old parameters can then be recalled as needed to solve previously learned tasks. Figure 1 provides an overview of our proposed framework. Our system uses a fraction of the space needed for storing individual networks, while retaining excellent performance across all learned tasks. Our method is loosely inspired by the role that the hippocampus is purported to play in memory consolation (Teyler and DiScenna, 1986). As noted in Preston and Eichenbaum (2013), during learning the brain forms an initial neural representation in cortical regions. The hippocampus then consolidates this representation into a form that is optimized for storage and retrieval. Specifically, there is evidence that the hippocampus uses experience replay (ER) to perform this knowledge consolidation (Carr et al., 2011; Kumaran et al., 2016). Traditionally, this observed phenomenon of repeated neural activity on compressed time-scales has been interpreted, and in some cases empirically validated, as the brain replaying past experiences. Our approach uses a similar scheme, but instead of replaying environmental observations and experiences, we continually recall and reconstruct the learned synaptic weights themselves. In more detail, we propose a system that consists of three components: (1) a set of reusable *task-networks* (TNs), (2) a *Buffer* in which we store the latest *m* learned weights exactly, and (3) a lifelong *autoencoder* (AE) with which we can encode a very large number of older networks. The AE learns a low-dimensional representation for each of the high-dimensional parameter vectors that define the weights of the TNs. Thus, our system *self-models* its own behavior, allowing it to approximate previously learned parameters instead of storing them directly. After the compact latent representations have been learned, the original networks may be discarded. If our system needs to solve a previously learned task, it can generate an approximation of the original weights by feeding the corresponding latent vector through the AE and then loading the reconstructed weights onto a TN. Consequently, our approach leverages the flexibility of conventional neural networks while avoiding their inability to remember old tasks.

## 2. Prior Work

Several methods have recently emerged for continual learning in deep networks (Parisi et al., 2018a). However, existing approaches either (1) restrict new learning, (2) store a new network (or equivalent number of parameters) for each task, or (3) require old training data. Some methods combine two or more of the above strategies. Notable examples of the first type include Elastic Weight Consolidation (EWC) (Kirkpatrick et al., 2017), Synaptic Intelligence (Zenke et al., 2017), Variational Continual Learning (Nguyen et al., 2018) (which also reuses old data), Progress and Compress (Schwarz et al., 2018), and Learning without Forgetting (Li and Hoiem, 2016). These approaches reuse the same (or most of the same) network for each new task but apply a regularization method to restrict changes in weights over time. Since they maintain a single network, these methods typically use constant space^1^. However, weight regularization is very likely to hamper a network's ability to acquire new knowledge because it prevents the network from updating its weights based on the exact gradient derived from the current loss.

The second category includes Progressive Networks (Rusu et al., 2016), Dynamically Expandable Networks (Yoon et al., 2018), and Context-Dependent Gating (Masse et al., 2018). These methods often achieve excellent performance, but they must store a new network's worth of parameters for each new task. In addition, methods in this category usually also utilize either regularization or retraining on old data. This is because standard neural networks use all their parameters to compute an output. In other words, every parameter in every task must either (1) help estimate the correct output or (2) minimally interfere with the correct output. Thus, to prevent old and new parameter values from interfering with each other, these networks must either keep some old training data or restrict the old parameter values from changing. Their main advantage is in facilitating *transfer learning*, i.e., using previous learning to speed up new learning.

Third, some methods store a fraction of the old training data and use it to retrain the network on previously learned tasks. Key approaches include Experience Replay (Mnih et al., 2013; van de Ven and Tolias, 2018) iCarl (Rebuffi et al., 2016), Variational Continual Learning (Nguyen et al., 2018), and (Lopez-Paz and Ranzato, 2017). Unfortunately, this paradigm combines the drawbacks of the previous two. First, most of these methods use a single network, so they cannot continually learn a large number of tasks well. Second, their storage requirements grow very quickly with the number of tasks because they have to store old training data. Moreover, data usually takes up orders of magnitude more space than the network itself because a trained network is effectively a compressed representation of the training set (Doersch, 2016). A few methods reduce this storage requirement by storing a compressed representation of the data, including Lifelong Generative Modeling (Ramapuram et al., 2017), FearNet (Kemker and Kanan, 2018), Deep Generative Replay (Shin et al., 2017), and Expert Gates (Aljundi et al., 2017). Some methods specifically use autoencoders for this task (Zhou et al., 2012; Riemer et al., 2017; Parisi et al., 2018b). However, even when compressed, data requires significantly more parameters to store than parametric networks. Neural networks themselves can be considered compressed representations of the datasets upon which they have been trained, as long as the number of parameters in the network is smaller than the number of data points in the training set. Therefore, we believe that it makes more sense to continually compress the trained networks themselves, as opposed to compressing the data.

Very recently, there has been a flurry of interest in the potential benefits of storing many separate models, as opposed to a single model optimized over all tasks. These techniques include Continual Learning with HyperNetworks (von Oswald et al., 2019), Task Agnostic Continual Learning via Meta-Learning (He et al., 2019), Online Meta-Learning (Finn et al., 2019), and Deep Online Meta-Learning (Nagabandi et al., 2018). Although our idea bears some similarity to these approaches (e.g., storing different weights for different tasks), they share the common trait of requiring access to old data in order to facilitate continual learning, whereas our approach does not.

## 3. Problem Formulation

Continual learning is not a single problem, but a family of related problems, each of which imposes a different set of constraints on the learning process (e.g., fixed architecture, no access to prior training data, etc.). Here, we consider the setting in which (1) the system learns one new task at a time, (2) each task can be solved independently of other tasks, (3) tasks have labels (i.e., the system knows which task to solve at any point), and (4) the system has *no access* to old training data. In particular, our problem differs from settings in which a single task grows more difficult over time [e.g., class-incremental learning (CIL)].

More concretely, each task *T*_*i*_ is specified by a training set, *D*_*i*_ = {*X*_*i*_, *Y*_*i*_}, consisting of *n*_*i*_ different {*x, y*} training pairs. The system is sequentially trained on each *D*_*i*_ dataset, using either a supervised or reinforcement learning paradigm, as applicable. That is, the system is first exposed to *D*_1_ (and thus must learn *T*_1_), then *D*_2_, *D*_3_, up to *D*_*k*_, where *k* is the total number of tasks encountered during its lifetime. Note that, in this paradigm, datasets are *not* required to be disjoint, i.e., any two datasets *D*_*i*_ and *D*_*j*_ many contain some common {*x, y*} pairs.

Critically, the system is trained on each *D*_*i*_ only once during its lifetime. The system is not allowed to store any exemplars from previous tasks or revisit old data when training on new tasks. We do, however, allow multiple passes over the data when first learning the task, as is standard in machine learning. We also assume that task labels are known; inferring the desired task from the input data is important but is outside the scope of this paper.

As noted in section 2, there are two common types of solutions for this CL problem. Regularization methods estimate a single set of parameters θ^*^ for all tasks, while growth-based approaches learn (and store) a new set of weights θ_*i*_ for each new task. The former uses constant storage (w.r.t to the number of tasks) but has bad performance, while the latter achieves good performance but is asymptotically equivalent to storing independent networks. Below, we detail our proposed approach, which has nearly the same performance as growth-based methods, but uses significantly less storage.

## 4. Methodology

Figure 1 provides a high-level overview of our proposed approach. Our Self-Net system uses a set of *c* reusable task-networks (TNs), an *m*-dimensional Buffer for storing newly learned tasks, and an *O*(*n*) lifelong autoencoder (AE) for storing older tasks. In addition, we store an *s*-dimensional latent vector for each task, where *s* << *n*. Assuming that *c* and *m* are constants, our space complexity is *O*(*n* + *ks*), where *k* is the number of learned tasks. In particular, our approach achieves asymptotic space savings compared to storing *kn* independent networks if *s* is sub-linear w.r.t. *n*, [i.e., *s* = ω(*n*) in asymptotic notation].

Each TN is just a standard neural network, which can learn regression, classification, or reinforcement learning tasks (or some combination of the three). For ease of discussion, we will focus on the case where there is a single TN and the Buffer can hold only one network; the extension to multiple networks and larger Buffers is trivial. The AE is made up of an *encoder* that compresses an input vector into a lower-dimensional, latent vector *e* and a *decoder* that maps *e* back to the higher-dimensional space. Our system can produce high-fidelity recollections of the learned weights, despite this intermediate compression. In our experiments, we used a contractive autoencoder (CAE) (Rifai et al., 2011) due to its ability to quickly incorporate new values into its latent space.

In CL, we must learn *k* different tasks sequentially. To learn these tasks independently, one would need to train and save *k* networks, with *O*(*n*) parameters each, for a total of *O*(*kn*) space. In contrast, we propose using our AE to encode each of these *k* networks as an *s*-dimensional latent vector, with *s* << *n*. Thus, our method uses only *O*(*n* + *ks*) space, where the *O*(*n*) term accounts for the TNs and the fixed-size Buffer. Despite this compression, our experiments show that we can obtain a high-quality approximation of previously learned weights, even when the number of tasks exceeds the number of parameters in the AE. Below, we first describe how to encode a single task-network before discussing how to encode multiple tasks in continual fashion.

### 4.1. Single-Network Encoding

Let *t* be a task (e.g., recognizing faces) and let θ be the *O*(*n*)-dimensional vector of parameters of a network trained to solve *t*. That is, using a task-network with parameters θ, we can achieve performance *p* on *t* (e.g., a classification accuracy of 95%). Now, let $\hat{\theta }$ be the approximate reconstruction of θ by our autoencoder and let $\hat{p}$ be the performance that we obtain by using these reconstructed weights for task *t*. Our goal is to minimize any performance loss w.r.t. the original weights. If the performance of the reconstructed weights is acceptable, then we can simply store the *O*(*s*) latent vector *e*, instead of the *O*(*n*) original vector θ.

If we had access to the test data for *t*, we could assess this difference in performance directly and train our AE until we achieved an acceptable margin ϵ:

(1) $$\begin{array}{r} p-\hat{p}\leq \epsilon . \end{array}$$

For example, for a classification task we could stop training our AE if the drop in accuracy is less than 1%.

In a continual learning setting, though, the above scheme requires storing validation data for each old task. Instead, we measure a distance between the original and reconstructed weights and stop training when we achieve a suitably close approximation. Empirically, we determined that the cosine similarity,

(2) $$\begin{array}{r} \cos\left(\theta ,\hat{\theta }\right)=\frac{\theta \cdot \hat{\theta }}{∥\theta ∥∥\hat{\theta }∥}=\frac{\sum_{i=1}^{n}\theta_{i}{\hat{\theta }}_{i}}{\sqrt{\sum_{i=1}^{n}\theta_{i}^{2}}\sqrt{\sum_{i=1}^{n}{\hat{\theta }}_{i}^{2}}}, \end{array}$$

is an excellent proxy for a network's performance. Unlike the mean-squared error, this distance metric is scale-invariant, so it is equally suitable for weights of different scales, which may be the case for separate networks trained on distinct tasks. As detailed in section 5, cosine similarity close to 0.99 yielded excellent performance for a wide variety of tasks and architectures.

### 4.2. Continual Encoding

We will now detail now to use our Self-Net to encode a sequence of trained networks in a continual fashion. Let *m* be the size of the Buffer, and let *k* be the number of tasks which have been previously encountered. As noted above, we train each of these *m* task-networks using conventional backpropagation, one per task. Now, assume that our AE has already learned to encode the first *k* task-networks. We will now show how to encode the most recent batch of *m* task-networks corresponding to tasks {*t*_*k*+1_, …, *t*_*k*+*m*_} into compressed representations {*e*_*k*+1_, …, *e*_*k*+*m*_} while still remembering all previously trained networks.

Let *E* be the set of latent vectors for the first *k* networks. In order to integrate *m* new networks {θ_*k*+1_, …, θ_*k*+*m*_} into the latent space, we first recollect all previously trained networks by feeding each *e* ∈ *E* as input to the decoder of the AE. We thus generate a set *R* of recollections, or approximations, of the original networks (see Figure 1). We then append each θ_*i*_ in the Buffer to *R* and retrain the AE on all *k*+*m* networks until it can reconstruct them, i.e., until the average of their respective cosine similarities is above the predefined threshold. Algorithm 1 summarizes our CL strategy.

As our experiments show, our compressed representations achieve excellent performance compared to the original parameters. Since each $\hat{\theta }\in R$ is simply a vector of network parameters, it can easily be loaded back onto a task-network with the correct architecture. We can thus discard the original networks and store *k* networks using only *O*(*n* + *ks*) space. In addition, our framework can encode many different types and sizes of networks in a continual fashion. In particular, we can encode a network of arbitrary size *q* using a constant-size AE (that takes inputs of size *n*) by splitting the input network into *r* subvectors^2^, such that (*n* = *q*/*r*). As we verify in section 5, we can effectively reconstruct a large network from its subvectors and still achieve a suitable performance threshold.

**Figure 2:**
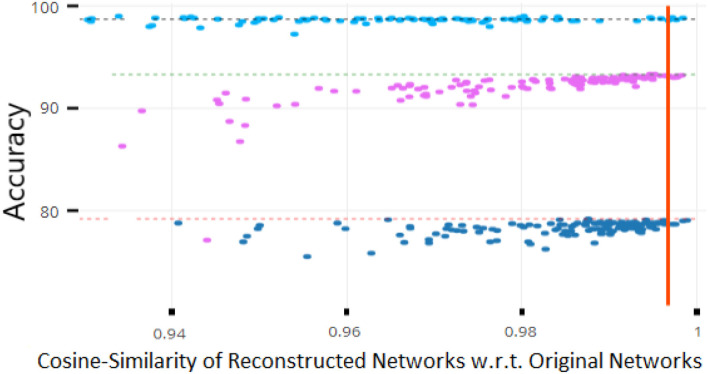
Robustness analysis of network performance as a function of cosine similarity. Each dot represents the accuracy of a reconstructed network and the dotted lines are the baseline performances of the original networks. The above values for three datasets Permuted MNIST (in pink), MNIST (in cyan), and CIFAR-10 (in blue), show that cosine similarity values above 0.997 guarantee nearly optimal performance for these datasets.

As Figure 2 illustrates, we empirically found a strong correlation between a reconstructed network's performance and its cosine similarity w.r.t. to the original network. Intuitively, this implies that vectors of network parameters that have a cosine similarity approaching 1 will exhibit near-identical performance on the underlying task. Thus, the cosine similarity can be used as a terminating condition during retraining of the AE. In practice, we found a threshold of 0.997 to be sufficient for most experiments.

#### 4.2.1. Autoencoder Details

Our proposed framework is agnostic to the choice of autoencoder. However, in our experiments we used contractive autoencoders (CAE) (Rifai et al., 2011) because we empirically found them to be more robust than other types of AEs, including variational autoencoders (Doersch, 2016). CAEs are identical to standard AEs, except that their loss function penalizes changes to the latent vector's values:

(3) $$\begin{array}{r} CAE_{loss}\left(\theta \right)=\cos\left(\theta ,\hat{\theta }\right)+\lambda ∥J_{f}\left(\theta \right){∥}_{F}^{2}. \end{array}$$

The first term is the cosine similarity discussed above (see Equation 2), while the regularization term is given by the Frobenius norm of the Jacobian w.r.t to each training input *x*_*i*_:

(4) $$\begin{array}{r} ∥J_{f}\left(x\right){∥}_{F}^{2}=\underset{ij}{\sum }(\frac{\partial h_{j}\left(\theta \right)}{\partial x_{i}}{)}^{2}. \end{array}$$

where *h*_*j*_(θ) are the parameters for the *j*-th hidden unit. In our experiments, we used a value of 0.0001 for lambda.

#### 4.2.2. Task Network Fine-Tuning

As an additional optimization, one can improve the speed with which the AE learns a new task by encouraging the parameters of new task-networks to be are as similar as possible to previously learned ones. This can be accomplished by fine-tuning all networks from a common source and penalizing large deviations from this initial configuration with a regularization term. Note that training new task networks in this manner differs from standard regularization methods (e.g., EWC; Kirkpatrick et al., 2017) because the weights learned for older tasks are not modified (and hence their performance does not degrade).

Formally, let θ^*^ be the source parameters, ideally optimized for some highly-related task. Without loss of generality, we can define the loss function of task-network θ_*i*_ for task *t*_*i*_ as:

(5) $$\begin{array}{r} TaskNetLoss_{i}=TaskLoss+\lambda MSE\left(\theta^{*},\theta_{i}\right) \end{array}$$

where λ is a regularization coefficient that determines the importance of remaining close to the source parameters vs. optimizing for the current task. By encouraging the parameters for all task-networks to remain close to one another, we make it easier for the AE to learn a low-dimensional representation of the original space. We employ this scheme for the experiments in section 5.2, with λ = 0.001.

## 5. Experimental Results

We carried out a range of CL experiments on a variety of datasets, in both supervised and reinforcement-learning (RL) settings. First, we performed two robustness analyses: (1) we empirically established how precise an approximation of a network must be in order to retain comparable performance on a task, and (2) we verified that our approach can reconstruct multiple networks trained on the same task. Then, we analyzed our system's ability to encode a very large number of tasks, thus validating that the AE does simply memorize the TNs. We then evaluated the performance of our approach on the following CL datasets: Permuted MNIST (Kirkpatrick et al., 2017), Split MNIST (Nguyen et al., 2018), Split CIFAR-10 (Zenke et al., 2017), Split CIFAR-100 (Zenke et al., 2017), and successive Atari games (Mnih et al., 2013) (we describe each dataset below). Finally, we also analyzed our system's performance when using **(2)** different sizes of AEs, and **(3)** different TN architectures.

**Algorithm 1:**
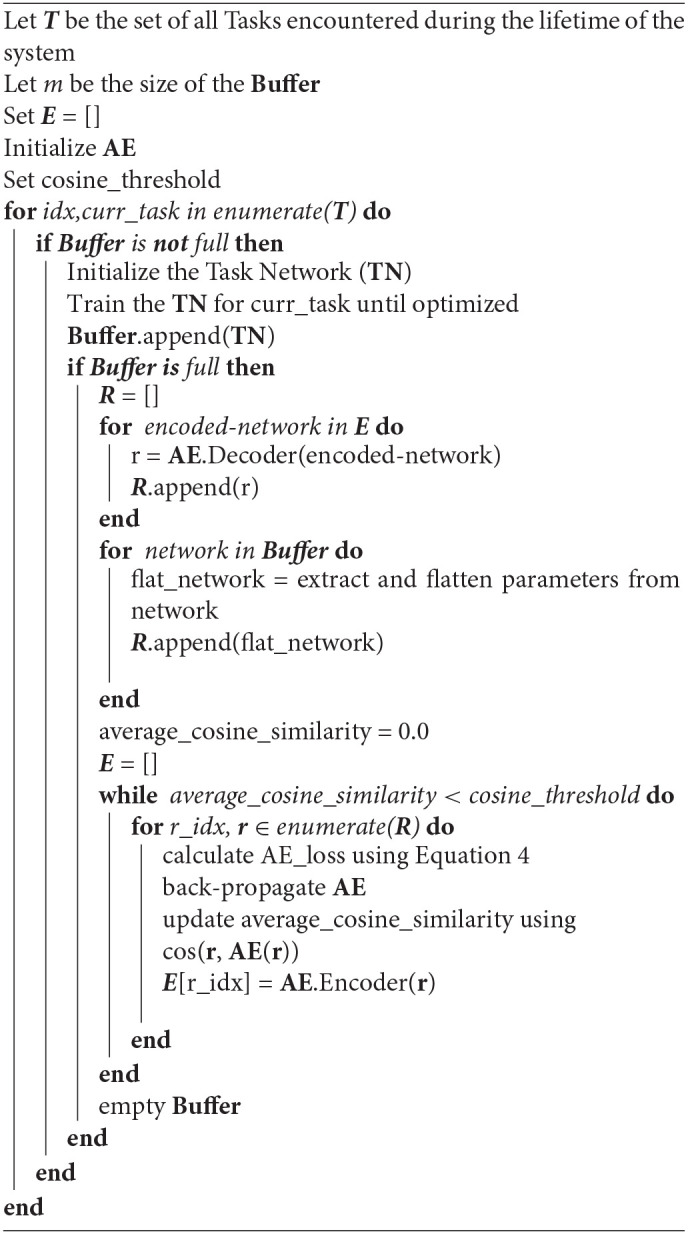
Lifelong learning via continual self-modeling

### 5.1. Robustness Analysis

#### 5.1.1. Parameter Noise Tolerance

Since the reconstructions performed by our autoencoder are approximate, our technique requires the trained weights of a neural network to be robust to certain levels of noise. In other words, the task network must retain its ability to properly map inputs to outputs, even if those weights are slightly perturbed (Blundell et al., 2015). To empirically validate how robust our task network's weights need to be, we added different levels of i.i.d, zero-mean Gaussian noise to the weights of a trained network. Our goal was two-fold: (1) to verify that approximate weights can differ from their original values while still retaining good performance and (2) to establish a threshold at which to stop training our AE. Since we assume no access to data from previously learned tasks, we need a way to estimate the performance of a reconstructed network without testing on a validation set.

Figure 2 shows performance as a function of deviations from the original parameters as measured by cosine similarity, for three datasets (described below). Under this metric, there is a clear correlation between the amount of parameter dissimilarity and the probability of a decrease in performance. The red line indicates a cosine similarity of 0.997. Weights above this value had nearly identical performance to the original values. Thus, unless otherwise noted, we used this threshold as a terminating condition in our subsequent experiments.

#### 5.1.2. Same-Task Reconstruction

In general, there are many possible weight configurations which yield identical input/output mappings. Therefore, in our second set of experiments, we verified that our approach can reconstruct different networks trained on the same task. First, we trained ten tasks networks on the MNIST dataset (LeCun et al., 1998), all initialized with the same initial values. Each network had two convolution layers (kernels of size 5 × 5, and stride 1 × 1), 1 hidden layer (320 × 50), and 1 output layer (50 × 10). Our corresponding AE had two, fully connected layers with 21,840 and 5 nodes, respectively. Second, we performed the same experiment on the CIFAR-10 dataset (Krizhevsky, 2009). Here, our task networks had two convolutional layers, followed by three fully connected hidden layers, and a final layer having 2 output units (60 K parameters in total). Our CIFAR-10 AE had 62,006 in the input and output layers and 5 nodes in the hidden layer.

**Figure 3:**
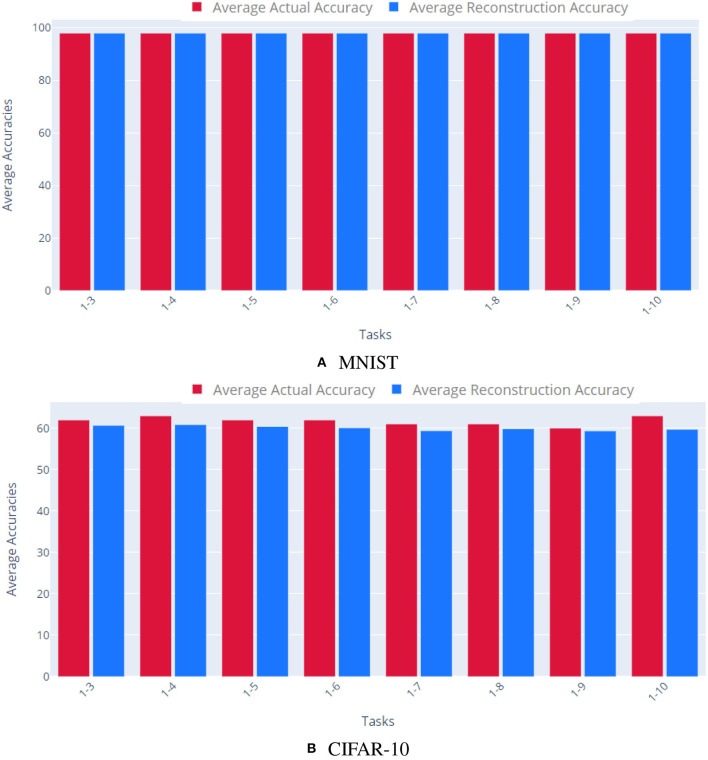
Reconstructing multiple networks for the same task. We trained 10 different networks independently to solve the same task and quantified our method's ability to reconstruct each task network. The top plot shows results for MNIST, the bottom plot for CIFAR-10. Each bar shows the cumulative average accuracy of all the tasks learned up to that point. Red bars indicate the accuracy of the original task networks, while blue show the accuracy of the reconstructed networks. For both datasets, the reconstructed networks achieve comparable accuracy to the original networks. **(A)** MNIST, **(B)** CIFAR-10.

As Figure 3 shows, our approach was able to reconstruct multiple, distinct networks trained on the same task. The AE accurately encoded the various parameter vectors, enabling the reconstructed networks to retain comparable performance to that of the original parameters for both MNIST and CIFAR-10.

### 5.2. Performance and Storage Scalability

In the next set of experiments, we verified that our method retains excellent performance even when the number of TN parameters exceeds the number of parameters in the AE. In other words, here we confirmed that our AE is *compressing previously learned weights*, not simply memorizing them. More generally, there is a trade-off in CL between storage and performance. Using different networks for *k* tasks yields optimal performance but uses *O*(*kn*) space, while regularized methods such as Online EWC (Huszár, 2018) only require *O*(*n*) space but suffer a steep drop in performance as the number of tasks grows. For any method, we can quantify performance as a *compression factor*, i.e., the number of additional parameters it stores per task; in our case, our compression factor is *k*/*s* because we store an *s*-dimensional vector per task.

Here, our experimental paradigm was as described in section 4.2: we first trained the TN on *m* tasks independently, storing each set of learned weights in the Buffer. Once the Buffer became full, we trained the AE to encode these weights into its latent space, only storing the latent vectors after training. We then continued to train the TN on new batches of *m* tasks (saving the new weights to the Buffer). Every time the Buffer became full, we trained the AE on *all tasks*, using the stored latent vectors and the new *m* weights. After the initial batch, we fine-tuned all networks from the mean of the initial set of *m* networks and penalized deviations from this source vector (using λ = 0.001), as described in section 4.

For these experiments, we used the Split MNIST dataset (Nguyen et al., 2018), which consists of different binary subsets of the MNIST dataset (LeCun et al., 1998), drawn randomly. In other words, tasks were defined by tuples comprised of the positive and negative digit class(es), e.g., ([pos={1}, neg={6,7,8,9}], [pos={6}, neg={1,2,3,4}], etc.). Here, the training and test sets consisted of approximately 40% positive examples and 60% negative examples. For this experiment, we trained a deep convolutional task network with two convolution layers (kernels of size 5 × 5 and stride 1 × 1), 1 hidden layer (320 × 50), and 1 output layer (50 × 10)—21,840 parameters in total. Our task network used ReLU activation units. Our AE, on the other hand, had one fully connected hidden layer with either 5 or 10 units. We used a Buffer of the same size as the latent vector, i.e., either 5 or 10. These values were chosen so that each new batch of networks yielded an integer compression factor, e.g., encoding 15 networks with a latent vector of size 5 gives 3X compression (*k*/*s* = 3). We used decreasing thresholds to stop training our AE: 0.9996 for the initial batch, 0.987 for the second batch, and 0.986 for subsequent batches.

**Figure 4:**
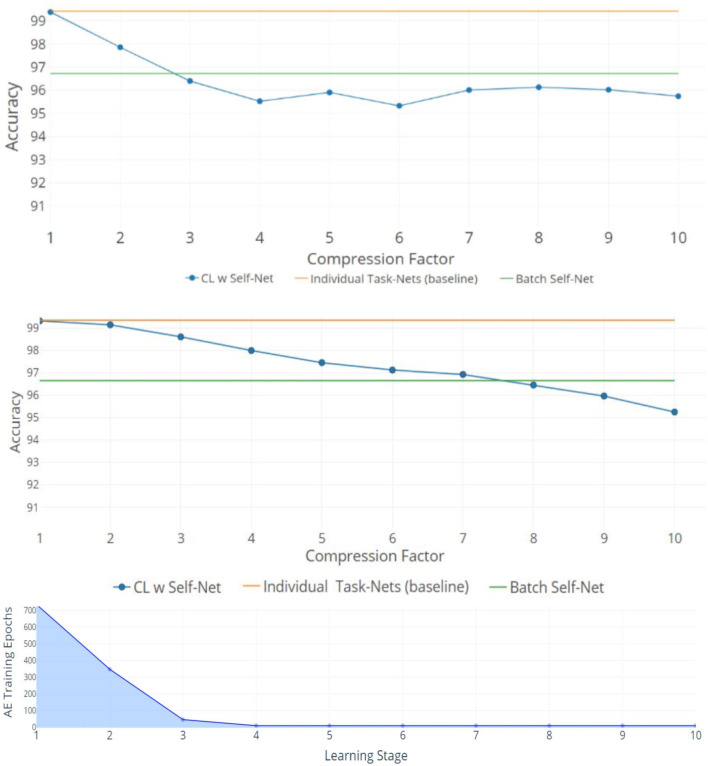
10X Compression for Split-MNIST. Orange lines denote the average accuracy achieved by individual networks, one per task. Green lines denote the average accuracy when training the AE to encode all networks as a single batch. Blue lines indicate the average accuracy obtained by Self-Net at each CL Stage. **(Top)** Fifty tasks with latent vectors of size 5 and a Buffer of size 5. **(Middle)** One hundred tasks with latent vectors of size 10 and Buffer of size 10. The x-axis **(top and middle)** denotes the compression factor achieved at each learning stage. **(Bottom)** The training epochs required by the five-dimensional AE to incorporate new networks decreases rapidly over time.

**Figure 5:**
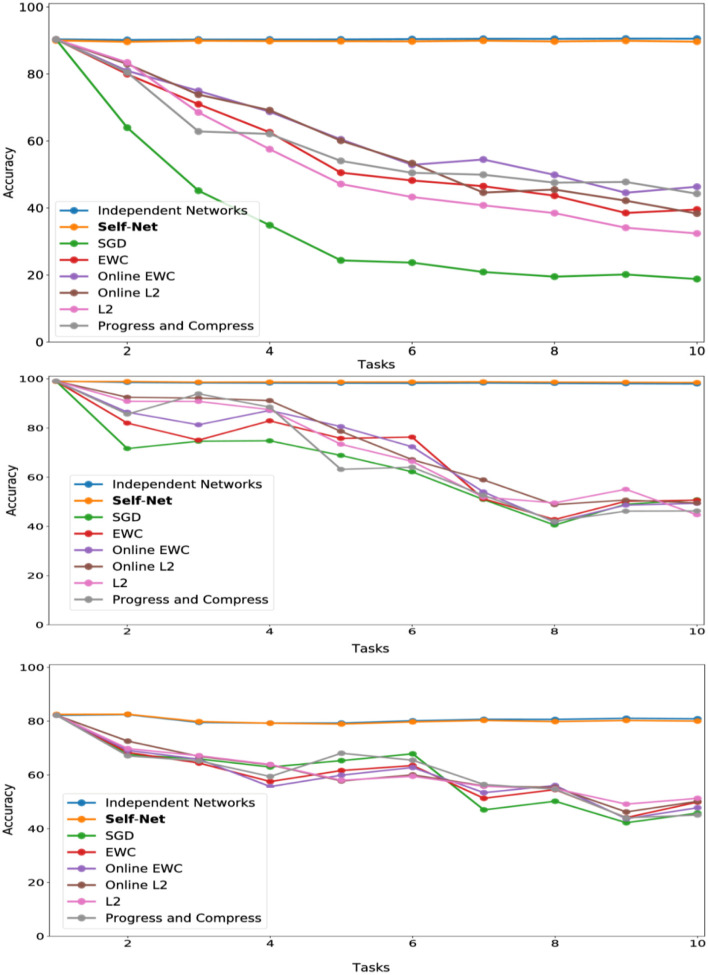
CL performance comparisons with average test set accuracy on all observed tasks at each stage for **(top)** Permuted MNIST, **(middle)** Split MNIST, and **(bottom)** Split CIFAR-10.

The top two plots of Figure 4 show the mean performance for up to 50 and 100 Split-MNIST tasks, given latent vectors of size 5 and 10, respectively. All figures show the average accuracy across all tasks learned up to that point. For comparison, we also plotted the original networks' performance and the performance of the reconstructions when the AE learned all the tasks in a single batch (green and orange lines, respectively). The line with dots represents the CL system; each dot indicates the point where the AE had to encode a new set of *m* networks. For 10X compression, the Self-Net with a latent vector of size 5 retained ~95.7% average performance across 50 Split-MNIST tasks, while the Self-Net with 10-dimensional latent vectors retained ~95.2% across 100 tasks. This represents a relative change of only ~3.3% compared to the original performance of ~99%. In other words, our approach is able to compress 21,840 parameters into 5 or 10 values with little performance loss, even when trained in a continual fashion. In contrast, existing methods dropped to ~50% performance after learning only 10 tasks on this dataset (see Figure 5 below). Finally, we note that by initializing each new network from the mean of the initial batch, our AE was able to incorporate subsequent networks with very little additional training (see stages 4–10 in bottom image of Figure 4).

### 5.3. Permuted MNIST

In the next set of experiments, we compared our approach to state-of-the-art methods across multiple datasets. First, we trained convolutional feed-forward neural networks with 21,840 parameters on successive tasks, each defined by distinct permutations of the MNIST dataset (LeCun et al., 1998), for 10-digit classification. We used networks with two convolution layers (kernels of size 5 × 5, and stride 1 × 1), 1 hidden layer (320 × 50), and 1 output layer (50 × 10). Our AE had three, fully connected layers with 21,840, 2,000, and 20 parameters, respectively. Thus, our latent vectors were of size 20. For this experiment, we used a Buffer of size 1. Each task network was encoded by our AE in sequential fashion, and the accuracies of all reconstructed networks were examined at the end of each learning stage (i.e., after learning a new task). Figure 5 (top) shows the mean performance after each stage for all tasks learned up to that point. Our technique almost perfectly matched the performances achieved by independently trained networks, and it dramatically outperformed other state-of-the-art approaches including EWC (Kirkpatrick et al., 2017), Online EWC (the correction to EWC proposed in Huszár, 2018), and Progress and Compress (Schwarz et al., 2018). As a baseline, we also show the results for SGD (no regularization), L2-based regularization in which we compare new weights to all the previous weights, and Online L2, which only measures deviations from the weights learned in the previous iteration. Our technique remember old tasks without inhibiting new learning.

### 5.4. Split MNIST

We then compared our method to the same set of prior approaches on the Split MNIST (described above). Our task-networks, CAE, and Buffer size were the same as for Permuted MNIST (except that the outputs of the task-networks were binary, instead of 10 classes). In this domain, too, our technique dramatically outperformed competing approaches, as seen in Figure 5 (middle)].

### 5.5. Split CIFAR-10

We then verified that our proposed approach could reconstruct larger, more sophisticated networks. Similar to the Split MNIST experiments above, we divided the CIFAR-10 dataset (Krizhevsky, 2009) into multiple training and test sets, yielding 10 binary classification tasks (one per class). We then trained a task-specific network on each class. Here, we used TNs having an architecture which consisted of two convolutional layers, followed by three fully connected hidden layers, and a final layer having two output units. In all, these task networks consisted of more than 60 K parameters. Again, for this experiment we used a Buffer of size 1. Our AE had three, fully connected layers with 20,442, 1,000, and 50 parameters, respectively. As described in section 4, we split the 60 K networks into three subvectors to encode them with our autoencoder; by splitting a larger input vector into smaller subvectors, we can encode networks of arbitrary sizes. The individual task-networks achieved accuracies ranging from 78 to 84%, and a mean accuracy of approximately 81%. Importantly, we encoded these larger networks using almost the same AE architecture as the one used in the MNIST experiments. As seen in Figure 5 (bottom), the accuracies of the reconstructed CIFAR networks also nearly matched the performances of their original counterparts, while also outperforming all other techniques.

### 5.6. Split CIFAR-100

**Figure 6:**
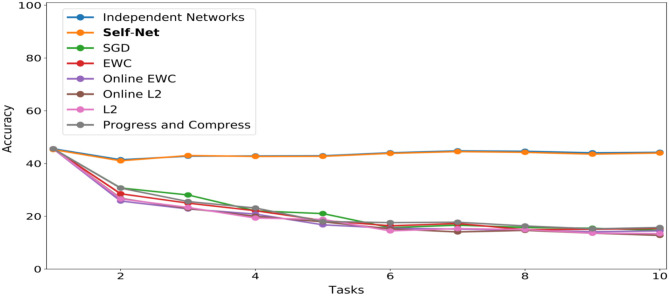
CL performance comparisons with average test set accuracy on all observed tasks at each stage for CIFAR-100.

We applied a similar approach for the CIFAR-100 dataset (Krizhevsky, 2009). That is, we split the dataset into 10 distinct batches comprised of 10 classes of images each. We used the same task-network architecture and Buffer size as in our CIFAR-10 experiments, modified slightly to accommodate a 10-class classification objective. The trained networks achieved accuracies ranging from 46 to 49%. We then encoded these networks using the same AE architecture described in the previous experiments, again accounting for the input size discrepancy by splitting the task-networks into smaller subvectors. As seen in Figure 6, our technique almost perfectly matched the performances achieved by independently trained networks.

### 5.7. Incremental Atari

To evaluate the CL performance of Self-Net in the challenging context of reinforcement learning, we used the code available at Greydanus (2017) to implement a modified Async Advantage Actor-Critic (A3C) framework; this architecture, originally introduced in Mnih et al. (2016), can learn successive Atari games while retaining good performance across all games. The model we used had four convolutional layers (kernals of size 3 × 3, and strides of size 2 × 2), a GRU layer (800 × 256), and two ouput layers: an Actor (256 × Num_Actions), and Critic (256 × 1), resulting in a complex model architecture and over 800 K parameters. Critically, this entire model can be flattened and encoded by the single AE in our Self-Net framework having three, fully connected layers with 76,863, 2,000, and 200 parameters, respectively. For these experiments we also used a Buffer of size 1.

**Figure 7:**
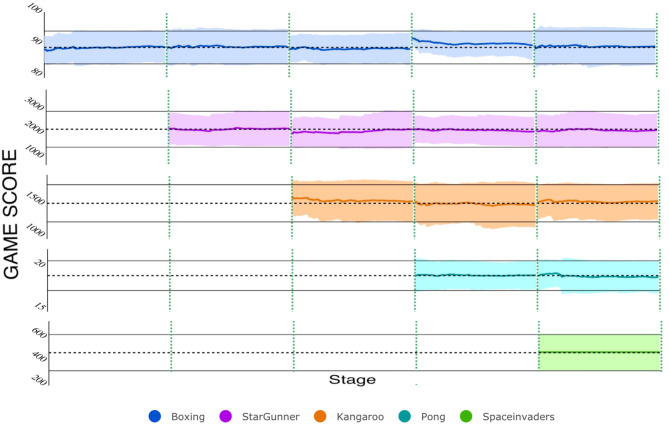
CL on five Atari games with Self-Net. To evaluate the reconstruction score at each stage, we ran the reconstructed networks for 80 full game episodes. The colored lines and bands represent the running mean and standard deviation in game score per episode. The cumulative mean score is nearly identical to the original TN at each stage.

Similar to previous experiments, we trained our system on successive tasks, specifically the following Atari games: Boxing, Star Gunner, Kangaroo, Pong, and Space Invaders. Figure 7 shows the near-perfect retention of performance on each of the five games over the lifetime of the system. This was accomplished by training on each game only once, never revisiting the game for training purposes. The dashed, vertical lines demarcate the different stages of continual learning. That is, each stage indicates that a new network was trained for a new game, over 40M frames. Afterwards, the mean (dashed, horizontal black lines) and standard-deviation (solid, horizontal black lines) of the network's performance were computed by allowing it to play the game, unrestricted, for 80 episodes. After each stage, the performances of all reconstructed networks were examined by re-playing each game with the appropriate reconstructed network. As Figure 7 shows, the cumulative means and SD's of the reconstructed networks closely mimic those achieved by their original counterparts.

### 5.8. Task-Incremental CORe50

**Figure 8:**
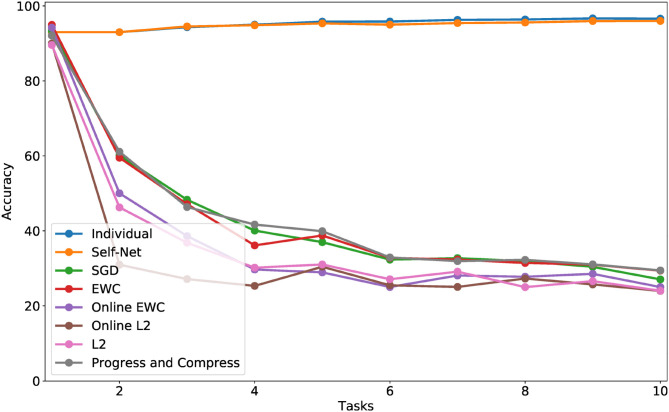
CL performance comparisons with average test set accuracy on all observed tasks at each stage for CORe50. Each observed task consisted of learning one new category (five classes).

To further validate that our idea scales to more challenging scenarios, we evaluated Self-Net on incremental tasks drawn from the CORe50 dataset (Lomonaco and Maltoni, 2017), which is comprised of 50 classes of objects, evenly divided between 10 categories (five classes per category). This dataset has 164,866, 128 × 128 color images in total, and it was originally constructed to measure the performance of deep learning systems on the difficult problem of class-incremental learning (CIL), a specific type of continual learning in which either new instances (NI), new classes (NC), or both (NIC) have to be learned over time. In this work, we consider learning new tasks (NT) over time, instead. This variant of CL (defined in section 3) is different from CIL, but it is still possible to use CORe50 to measure NT learning. Specifically, we trained Self-Net to sequentially learn five classes (a category) from the CORe50 dataset (10 tasks total). Given the larger image size of this dataset, the task network had over 140 K parameters. Our AE had three, fully connected layers with 48,813, 500, and 30 nodes, respectively. As described in section 4, we split the 140 K networks into three subvectors to encode them with our autoencoder. As Figure 8 shows, regularization methods such as EWC had comparable performance to standard SGD on this dataset, confirming that it is more challenging than MNIST or CIFAR-10. However, our proposed method almost perfectly matched the classification accuracies achieved by the original task networks (which ranged from 91 to 99), thus confirming that our approach is viable even in more difficult scenarios.

### 5.9. Split Networks and Multiple Architectures

**Figure 9:**
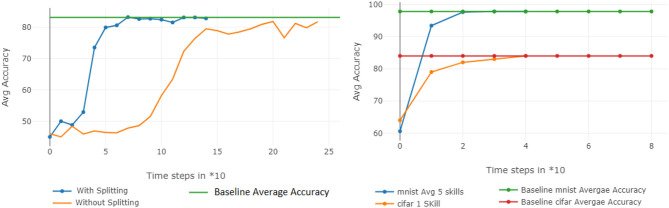
Additional analyses. **(Left)** The AE training efficiency is improved when large networks are split into smaller subvectors. **(Right)** A single AE can encode networks of different architectures and sizes.

Finally, we verified that (1) a smaller AE can encode multiple network splits in substantially less time than a larger one can learn the entire network and (2) that the same AE can be used to encode trained networks of different sizes and architectures. Figure 9 (left) shows the respective training rates of an AE with 20,000 input units (blue line)—trained to reconstruct 3 sub-vectors of length 20,000—compared to that of a larger one, with 61,000 input units (yellow line), trained on a single 60 K CIFAR-10 network. Clearly, using more inputs for a smaller AE enables us to more quickly encode larger networks. Finally, Figure 9 (right) shows that the same AE can simultaneously reconstruct 5 MNIST networks and 1 CIFAR network so that all networks approach their original accuracies.

## 6. Conclusions and Future Work

In this paper, we introduced a scalable approach for multi-context continual learning that decouples how to learn a set of parameters from how to store them for future use. Our proposed framework uses state-of-the-art autoencoders to facilitate lifelong learning via continual self-modeling. Our empirical results confirm that our method can efficiently acquire and retain large numbers of tasks in continual fashion. In future work, we plan to further improve our autoencoder's capacity and explore how to use the latent space to extrapolate to new tasks using little or no training data. We also intend to compress the latent space even further (e.g., using only log(*k*) latent vectors for *k* tasks). Promising approaches include clustering the latent vectors into sets of related tasks or using sparse latent representations. Finally, we will also investigate how to infer the current task automatically.

## Data Availability Statement

The source code and datasets analyzed for this study can be found in the following Github repository: https://github.com/jmandivarapu1/SelfNet-Lifelong-Learning-via-Continual-Self-Modeling.

## Author Contributions

RE conceived of the presented idea. JM and BC carried out the experiments. JM, BC, and RE wrote the manuscript.

### Conflict of Interest

The authors declare that the research was conducted in the absence of any commercial or financial relationships that could be construed as a potential conflict of interest.

## Footnotes

^1^Although, as noted in Huszár (2018), standard EWC stores an *O*(*n*) set of Fisher weights for each task. The modified version proposed in Huszár (2018) does use constant space.

^2^We pad with zeros whenever *q* and *n* are not multiples of each other.
